# P-584. Mpox Knowledge, Attitudes, and Practices Among Public Health Professionals in Bangladesh

**DOI:** 10.1093/ofid/ofaf695.798

**Published:** 2026-01-11

**Authors:** Monzur M Patwary, Naimul Islam

**Affiliations:** Emory University, Atlanta, GA; Canadian University Of Bangladesh, Dhaka, Dhaka, Bangladesh

## Abstract

**Background:**

The recent global outbreak of mpox has highlighted the critical need for public health preparedness in managing zoonotic diseases. Public health professionals' knowledge, attitudes, and practices regarding mpox are crucial for effective outbreak control and public education. This study aims to assess these factors among public health professionals in Bangladesh, providing insights to enhance preparedness and response strategies for mpox and similar emerging threats.

**Methods:**

A cross-sectional study was conducted using a convenient sampling approach among 23 public health professionals across 11 organizations. Data was collected through structured surveys evaluating their familiarity with Mpox symptoms, transmission, preventive measures, and perceptions of organizational preparedness. Descriptive statistics and Chi-squared tests were applied to analyze the relationships between awareness levels and professional characteristics.

**Results:**

The study revealed variability in Mpox awareness. Only 4.3% of participants were very familiar with its symptoms, while preventive practices such as vaccination and isolation were inconsistently recognized. Organizational preparedness was rated as "not prepared" by 35% of respondents. Despite these gaps, 87% of participants believed affected individuals would receive support, reflecting a generally supportive attitude, though concerns about stigma and discrimination persisted.

**Conclusion:**

The findings underline considerable gaps in awareness and preparedness among public health professionals in Bangladesh. The reliance on convenient sampling highlights potential biases but also reflects the challenges in accessing a wider cohort. Organizational and individual readiness for Mpox and similar outbreaks require thorough, targeted training and awareness building initiatives.

**Disclosures:**

All Authors: No reported disclosures

